# Supplementary material for: Splice-Junction-Based Mapping of Alternative Isoforms in the Human Proteome
Source: Cell Rep. Author manuscript; Available in PMC 2020 Jan 15. (PMC6961840; doi:10.1016/j.celrep.2019.11.026)
Supplement: 2 [file NIHMS1546469-supplement-2.zip › DF1/PXD010154/Heart/Heart_36_ART3_IPEPFPLPAPGPVPVPGPK.pdf]

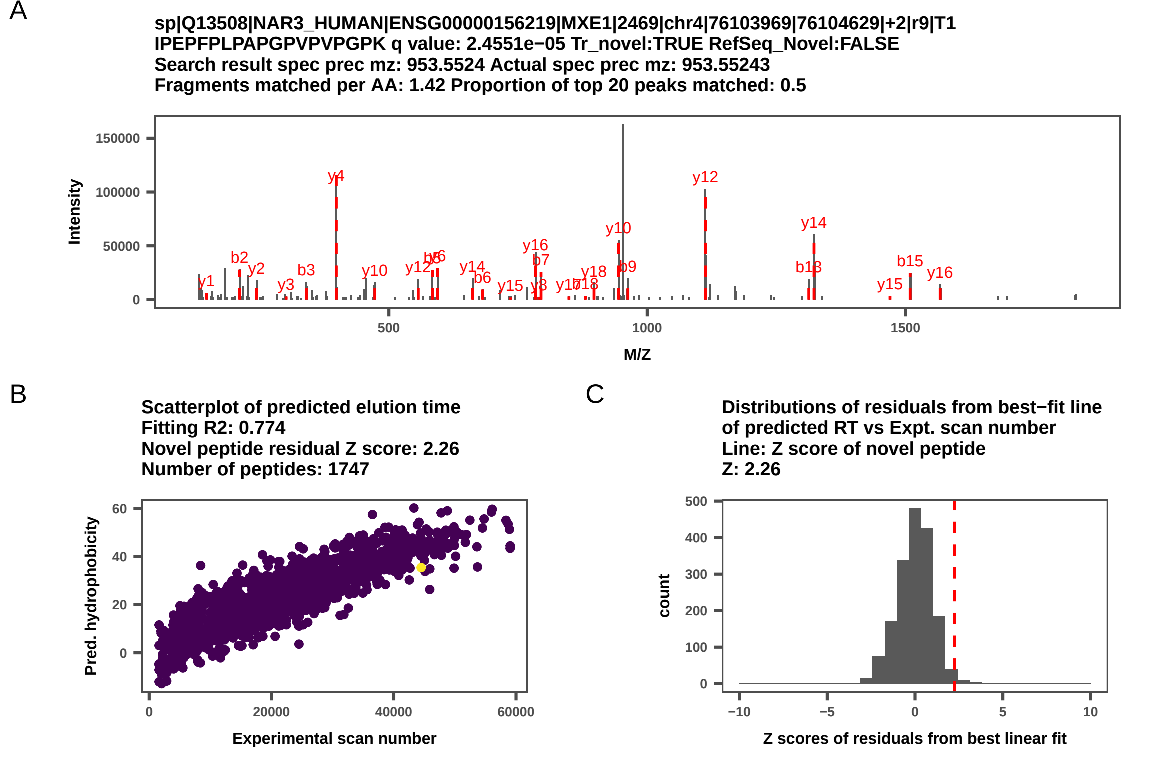
A
sp|Q13508|NAR3_HUMAN|ENSG00000156219|MXE1|2469|chr4|76103969|76104629|+2|r9|T1
IPEPFPLPAPGPVPVPGPK q value: 2.4551e−05 Tr_novel:TRUE RefSeq_Novel:FALSE
Search result spec prec mz: 953.5524 Actual spec prec mz: 953.55243
Fragments matched per AA: 1.42 Proportion of top 20 peaks matched: 0.5
Intensity
150000
100000
50000
0
500
1000
1500
M/Z
y1
b2
y2
y3
b3
y4
y10
y12
y14
b6
y15
y16
b7
y18
y10
b9
y12
y14
b15
y15
y16
B
Scatterplot of predicted elution time
Fitting R2: 0.774
Novel peptide residual Z score: 2.26
Number of peptides: 1747
Pred. hydrophobicity
60
40
20
0
0
20000
40000
60000
Experimental scan number
C
Distributions of residuals from best−fit line
of predicted RT vs Expt. scan number
Line: Z score of novel peptide
Z: 2.26
count
500
400
300
200
100
0
−10
−5
0
5
10
Z scores of residuals from best linear fit
